# A Stochastic Binary Model for the Regulation of Gene Expression to Investigate Responses to Gene Therapy

**DOI:** 10.3390/cancers14030633

**Published:** 2022-01-27

**Authors:** Guilherme Giovanini, Luciana R. C. Barros, Leonardo R. Gama, Tharcisio C. Tortelli, Alexandre F. Ramos

**Affiliations:** 1 Escola de Artes, Ciências e Humanidades, Universidade de São Paulo, Av. Arlindo Béttio, 1000, São Paulo 03828-000, SP, Brazil; ggiovanini@usp.br; 2 Centro de Investigação Translacional em Oncologia, Departamento de Radiologia e Oncologia, Faculdade de Medicina da Universidade de São Paulo, Instituto do Câncer do Estado de São Paulo, Av. Dr. Arnaldo, 251, São Paulo 01246-000, SP, Brazil; lucianalpt@gmail.com (L.R.C.B.); leonardo.gama@usp.br (L.R.G.); tharcisio.junior@hc.fm.usp.br (T.C.T.J.)

**Keywords:** epigenetic regulation in cancer treatment, stochastic binary regulation of gene expression, treatment targeting RKIP levels, reduction of heterogeneity of treatment response, gene therapy, multi-drug therapy

## Abstract

**Simple Summary:**

Gene editing technologies reached a turning point toward epigenetic modulation for cancer treatment. Gene networks are complex systems composed of multiple non-trivially coupled elements capable of reliably processing dynamical information from the environment despite unavoidable randomness. However, this functionality is lost when the cells are in a diseased state. Hence, gene-editing-based therapeutic design can be viewed as a gene network dynamics modulation toward a healthy state. Enhancement of this control relies on mathematical models capable of effectively describing the regulation of stochastic gene expression. We use a two-state stochastic model for gene expression to investigate treatment response with a switching target gene. We show the necessity of modulating multiple gene-expression-related processes to reach a heterogeneity-reduced specific response using epigenetic-targeting cancer treatment designs. Our approach can be used as an additional tool for developing epigenetic-targeting treatments.

**Abstract:**

In this manuscript, we use an exactly solvable stochastic binary model for the regulation of gene expression to analyze the dynamics of response to a treatment aiming to modulate the number of transcripts of a master regulatory switching gene. The challenge is to combine multiple processes with different time scales to control the treatment response by a switching gene in an unavoidable noisy environment. To establish biologically relevant timescales for the parameters of the model, we select the RKIP gene and two non-specific drugs already known for changing RKIP levels in cancer cells. We demonstrate the usefulness of our method simulating three treatment scenarios aiming to reestablish RKIP gene expression dynamics toward a pre-cancerous state: (1) to increase the promoter’s ON state duration; (2) to increase the mRNAs’ synthesis rate; and (3) to increase both rates. We show that the pre-treatment kinetic rates of ON and OFF promoter switching speeds and mRNA synthesis and degradation will affect the heterogeneity and time for treatment response. Hence, we present a strategy for reaching increased average mRNA levels with diminished heterogeneity while reducing drug dosage by simultaneously targeting multiple kinetic rates that effectively represent the chemical processes underlying the regulation of gene expression. The decrease in heterogeneity of treatment response by a target gene helps to lower the chances of emergence of resistance. Our approach may be useful for inferring kinetic constants related to the expression of antimetastatic genes or oncogenes and for the design of multi-drug therapeutic strategies targeting the processes underpinning the expression of master regulatory genes.

## 1. Introduction

Recent advances in gene editing technologies brought the promise of a turning point for gene therapy [1] toward more complex therapeutic designs aiming to orchestrate the expression of gene networks for cell phenotype reprogramming [2]. One possibility is to develop cancer treatment strategies to revert metastasis by targeting master regulatory genes [3]. Mathematical models describing the regulation of gene expression can be insightful for engineering of the dynamics of the gene networks governing cellular behavior.

Let us assume the ideal case in which the editing exclusively affects its epigenetic target [2] within tumor cells. The task can be formulated as a control problem to enable the number of transcripts of a master regulating gene to have its average value at a given level and random fluctuations within a sufficiently small range. The control may be performed by external agents, such as a combination of drugs that we would like to keep at a minimally effective dosage because of the eventual toxicity.

Despite our deepened understanding of cancer biology due to the advances in molecular biology techniques, the use of quantitative methods to integrate the plethora of generated data to design treatments targeting metastasis is still in its infancy [4,5]. The genotypic variability and intrinsic randomness of biochemical reactions [6] governing epigenetics underpin the multiplicity of cancer cell phenotypes commonly termed as tumor heterogeneity [7,8,9,10,11,12].

Additionally, cellular processes are controlled by several networks of chemical reactions characterized by changeable topologies and functional redundancies that equip cells with adaptation and robustness capabilities [13,14,15,16]. The numerous characteristic timescales of the chemical processes taking place inside the cell add another layer of cumbersomeness [17,18].

Thus, the enhancement of therapeutic strategies requires the analysis and engineering of the dynamics of treatment response in a complex system composed of several interacting components with a multiplicity of characteristic time scales and subjected to randomness. Finding building blocks with those features may provide useful insights on how to modulate the dynamics of such a complex system [19]. For that, the exactly solvable stochastic model for transcription of a binary gene [20,21,22,23] is a good candidate to be used as a prototype for simulating enhanced treatment strategies.

### Rationale

In this manuscript, we propose a *proof of concept* of a quantitative tool to investigate the response to the application of multiple drugs for epigenetic control of master regulatory genes. Those treatment designs involve a large number of chemical compounds with diverse half-lives and interacting affinities with the gene components. A gene may have its transcription guided by a promoter with multiple states, and the duration of those states may be regulated by multiple transcription factors with various affinities to the regulatory sites of the gene.

For the promoter in the ON state, one would still have variation on the affinity between PolII and the promoter. Additionally, one needs to select the typical external agents, here drugs, that will affect the processes involved in the modulation of gene expression accordingly with their pharmacokinetic parameters. Hence, mathematical modeling of treatment targeting specific genes requires the selection of a proper gene model system to enable the determination of the parameter values associated with the regulation of its expression. One needs to couple this model with the pharmacokinetics of the interaction of the drugs with their targets.

This can be formulated as a control problem in which we aim to keep both the average expression level of a target gene and the heterogeneity of its response to treatment within specific ranges while minimizing the amounts of drugs used to prevent toxicity. To devise a quantitative strategy to overcome the challenge above, we started with the simplest possible mathematical model for regulated gene expression under influence of randomness to establish a *proof of concept*. This stochastic binary model for gene expression has four parameters and two characteristic time scales. Ideally, each of the multiple drugs composing a treatment would affect a specific process (or mathematically speaking, parameter) involved in the expression of the target gene.

This implies on adding at least two parameters per drug: the halflife of the drug, and the strength of its effect in each specific parameter of the gene expression model. The next challenge is to orchestrate this multiplicity of parameters to properly regulate the expression of the target gene. Here, we analyze the drug dose and gene response using a “pedestrian optimization” as application of optimal control theory is a highly elaborate technique with specific requirements and the formulation is beyond the scope of the current study [24]. One advantage is that multiple qualitative features of the stochastic model for gene expression are preserved independently of the numerical values that are used.

Our next challenge is the selection of an appropriate candidate model system. Here, we use the gene encoding Raf kinase inhibitory protein (RKIP) because it plays a major role in regulating the dynamics of multiple components of a cell [25]. Since cancer lethality is mainly caused by metastasis, the choice of RKIP is promising as its concentrations are typically reduced in metastatic cancers [26,27,28]. This negative correlation turns RKIP and RKIP-related gene signatures into useful biomarkers of metastatic risk in cancer patients [29].

The characterization of RKIP as an anti-metastatic gene is reinforced by experiments demonstrating that its overexpression blocks in vivo invasion and metastatic progression [30,31]. Hence, we focus on possible strategies to increase the amounts of RKIP in cancer cells by re-modulating its expression profiles toward the pre-cancer regimens. In some cancers, the mean number of RKIP transcripts is similar to that of cells of a normal tissue, but the variability is significantly increased [32]. Our approach is also potentially useful for those cases because it is based on stochastic processes.

The selection of RKIP as a model system provides us with its degradation rate, i.e., one of the characteristic times of our stochastic model for regulation of gene expression. The additional characteristic time is the gene switching rate, which is usually unavailable because it needs to be inferred. Once the RKIP gene is selected, we may also consider a multi-drug treatment based on 5-AzaC and DETANONOate because they have been tested previously, their mechanisms of action are sufficiently known, and because we could recover their half-life times.

Hence, in the case of a treatment design with two drugs, we will have three out of four half-life times for the system composed by the genes and treatment. This prompts us to investigate the qualitative features of our prototypic model as an additional tool for simulating cancer treatment designs targeting specific genes (Note, however, that parameter value adjustments for specific experimental designs can be performed if one judges that our model may provide sufficiently useful results).

For that goal, we simulate the effect of application of multiple drugs each targeting a different kinetic rate of a two-state stochastic model for gene transcription. Due to the treatment, we will consider the kinetic rates of the model to be time-dependent in response to drug application. This enables one to estimate the speed and heterogeneity of response to treatment by, respectively, computing the dynamics of the average number of transcripts and their variance. The exact solutions of the model at constant kinetic rates have two characteristic time scales, one related with the promoter state switching and the second being the mRNA lifetime.

Recently, we demonstrated that the ratio between those two time scales may be used to classify the qualitatively distinguishable noise regimens of gene transcription on the binary model [23,33] and the reliability of information about the promoter state that is transmitted by gene products [34]. Here, we assume that the addition of a drug causes the kinetic rates of the model to become time dependent and that this effect decays exponentially such that it has up to four additional time scales for the problem. Then, we show that treatment targeting a gene with a fast switching promoter, and expressed as a quasi-Poissonian process, enables the fastest and least heterogeneous response to treatment.

If the gene has a slow switching promoter, the response will be slower and have higher heterogeneity. A gene expressed in a burst fashion will enable a fast response with maximal heterogeneity. Then, we build upon the previous analysis to design an enhanced treatment enabling a faster response with reduced heterogeneity that is independent of the pre-treatment time scales. The enhanced treatment is based on reduced drug dosages to reduce the chances of toxicity and emergence of resistance caused by compensatory effects [4,5].

The remainder of this manuscript is organized as follows. The assumptions underlying the qualitative and quantitative models that we propose are presented in Section 2. The obtained results are shown in Section 3, and we discuss them in Section 4. Our concluding remarks are presented in Section 5.

## 2. Methods and Models

Gene expression is the key mechanism in cell function by means of an extensive molecular machinery that transforms the genetic information into molecular functions. Gene expression can be described as a two-stage process—namely, the gene transcription that generates mRNA, and the mRNA translation, which has proteins as products. Here, we focus on gene transcription and provide a simplified picture of how it occurs in eukaryotic cells: the RNA Polymerase protein complex (RNAPolII) binds to a specific region of a gene, the promoter site, and begins transcription elongation.

The binding of RNAPolII to the promoter site may be regulated by its interaction with a DNA region, called an enhancer. The enhancers are regions of DNA that interact with the transcription factors that can provide a positive or negative regulation of the binding of RNAPolII to the promoter site. This is the process that we will use to model RKIP transcription.

### 2.1. A Brief Description of the Molecular Role of RKIP

RKIP protein is a regulator of kinases that directly binds to Raf kinase [32,35,36,37]. RKIP is involved in regulation of signaling pathways, such as the Raf–Mek–Erk cascade and the NF- κ B-related pathways [38,39], both participating in the regulation of anti-apoptosis processes. Additionally, those pathways modulate cell proliferation with the Raf–Mek–Erk cascade participating in differentiation and NF- κ B-related pathways acting in inflammation. In the Raf–Mek–Erk cascade, RKIP inhibits the downstream signaling pathway by the direct binding of the dephosphorylated RKIP protein to Raf-1.

This molecular complex prevents both Raf-1 phosphorylation and Raf-1/Mek association, which, in turn, causes the interruption of Erk signaling. This inhibition can be reverted by action of Protein Kinase C (PKC) [40,41,42], a post-translational regulator that phosphorylates RKIP. The latter becomes dissociated from Raf-1 because of its structural change, and the Erk pathway becomes activated. RKIP negatively regulates the NF- κ B signaling pathway indirectly [32,38] when it interacts with kinase complex IKK reducing their activity.

This causes a reduction in phosphorylation and degradation of the inhibitory proteins I κ B, which, in turn, inactivate NF- κ B. In addition, RKIP modulates other fundamental cell signaling pathways involving heterotrimeric G-proteins, keap1/nrf2, STAT3, and GSK [43]. Due to its interaction with multiple pathways, we consider RKIP as a master modulator of cellular processes.

The amounts of RKIP proteins inside the cell can be regulated at multiple steps of expression, from pre-transcription of the gene to post-translation [44]. A plethora of metastatic solid tumors have RKIP downregulated or lost, and the experimental data suggests that this happens because of transcriptional or post-transcriptional regulation [29]. Here, we limit our attention to cases in which a reduction in RKIP protein numbers happens because of repression of transcription.

Transcription of RKIP can be silenced by methylation of its promoter. Indeed, methylation-specific PCR (MSP) analysis has shown a sufficiently strong correlation between RKIP-promoter methylation and low RKIP expression levels in cancer tumors, [29], including esophageal and gastric [45,46]. Histone modifications are also found as epigenetic mechanisms to regulate RKIP levels.

Histone deacetylase inhibitors can increase RKIP transcripts [47,48]. Snail and BACH1 transcription factors can downregulate RKIP transcription by histone methyltransferases [49,50], and both are associated with the epithelial–mesenchymal transition. Snail is a direct transcriptional repressor of the gene encoding the cell adhesion protein E-cadherin [51], and BACH1 is the basic leucine zipper protein expressed in mammalian tissue, which positively regulates motility related genes that promote metastasis in breast cancers [52]. The expression of either Snail and BACH1 genes are self-repressed and repressed by RKIP proteins. Additionally, BACH1 and RKIP combine into a bistable gene circuit that describes a switch for metastatic phenotype in tumor cellular population, as recently shown [50].

RKIP mRNAs may also be post-transcriptionally regulated negatively because of the interaction with specific miRNAs. Indeed, in several cancers, the RKIP gene is silenced by the action of miRNAs (-224, -27a, -23a, and -543) targeting RKIP mRNAs [53,54]. This suggests a therapeutic alternative based on targeting miRNAs that downregulate RKIP expression, such as lncRNA XIST, which stabilizes RKIP expression by suppressing miR-23a [55].

The aforementioned data suggests an association between RKIP expression levels and tumor cell phenotypes. This leads to the possibility of using RKIP as a prognostic marker for survival probabilities [56,57]. Additionally, as RKIP levels are mainly reduced in several cancers [32], understanding its regulatory mechanisms [45,46,47,48,49,50,51,52] may enable the design of anti-metastatic treatments.

### 2.2. An Effective Model for the Regulation of Gene Expression

We interpret the gene as a source of gene products randomly switching between ON and OFF states. Synthesis of gene products takes place when the gene is ON at rate *k*, while, at OFF state, there is no synthesis. The rate of degradation of gene products is denoted by ρ . The gene switches from state ON to OFF, and from OFF to ON, with rates *h*, and *f*, respectively.

The aforementioned processes can be represented as a system of effective chemical reactions as given at Equations (1)–(4). We denote a gene product by P and its regulatory site by R . In this manuscript, we consider the particular case of positive regulation of the gene by a transcription factor denoted by $T_{F}$ .

(1) $$\begin{array}{ccc} {\mathrm{RT}}_{F} & \overset{k}{⇀} & {\mathrm{RT}}_{F}+P, \end{array}$$

(2) $$\begin{array}{ccc} P & \overset{\rho }{⇀} & ⊘, \end{array}$$

(3) $$\begin{array}{ccc} R+T_{F} & \overset{f}{⇀} & {\mathrm{RT}}_{F}, \end{array}$$

(4) $$\begin{array}{ccc} {\mathrm{RT}}_{F} & \overset{h}{⇀} & R+T_{F}. \end{array}$$

Equations (1) and (2) indicate, respectively, the gene product synthesis and degradation. The switching from OFF to ON state because of the binding of the activating protein, and the inverse transition caused by its unbinding, are, respectively, indicated in Equations (3) and (4). One may also consider the case of a transcription factor as a repressor. Then, the effective reactions Equations (3) and (4) denote the ON to OFF and OFF to ON state transitions with rates transformed as f→h and h→f .

This system of effective reactions is very simple if we consider the complexity of the regulation of gene expression in mammals. However, such a simplification enables the construction of exactly solvable quantitative models with a smaller number of parameters that we can use to investigate hypothetical treatment strategies before performing experiments. One example is the use of reduced dose multi-drug treatment targeting a functional network. A given drug has a specific half-life, and the multiple drugs that act on a system will combine as multi-timescale processes.

Combining the dosage and application agendas of those multiple drugs to ensure both effectiveness and non-toxicity may be a hard task, and a quantitative model may provide invaluable insights on the therapeutic design. Here, we use the stochastic binary model for the regulation of gene expression to investigate how the combination of two drugs modulates the dynamics of mRNA production.

### 2.3. An Effective Model for Investigating Regulation of Gene Expression Dynamics after Treatment

A biological interpretation of the effective model presented at Equations (1)–(4) is given. The rate *k* is proportional to the inverse of the time interval between two consecutive bindings of RNAPolII to the promoter site. This implies assuming that transcription starts after a negligible interval following RNAPolII binding. Alternatively, it might be interpreted as the inverse of the average interval between the initiation of two subsequent transcription processes.

The first case implies assuming the availability of large amounts of RNAPolII and interpreting *k* as a consequence of the affinity between the promoter and RNAPolII. The second could be interpreted as the efficiency of the promoter on initiating transcription. Those two interpretations are not exclusive, and, for simplicity, we refer to an increase in *k* as an increase in the efficiency of the promoter.

The rates of switching *h* and *f* are the inverse of the average time of availability and unavailability of the promoter for the binding of the RNAPolII, respectively. The value of those rates will be determined by the binding of transcription factors to the regulatory regions of the gene. Despite the amount of transcription factors that change with time, it is fair to assume that they will remain constant during sufficiently short intervals. Hence, during those short time intervals, we may employ the effective model of Equations (1)–(4). The values of the switching constants will reflect the balance resulting from the binding of activators, repressors, quenchers, pioneer factors, and other regulatory elements interacting with the regulatory regions of the gene.

**Figure 1:**
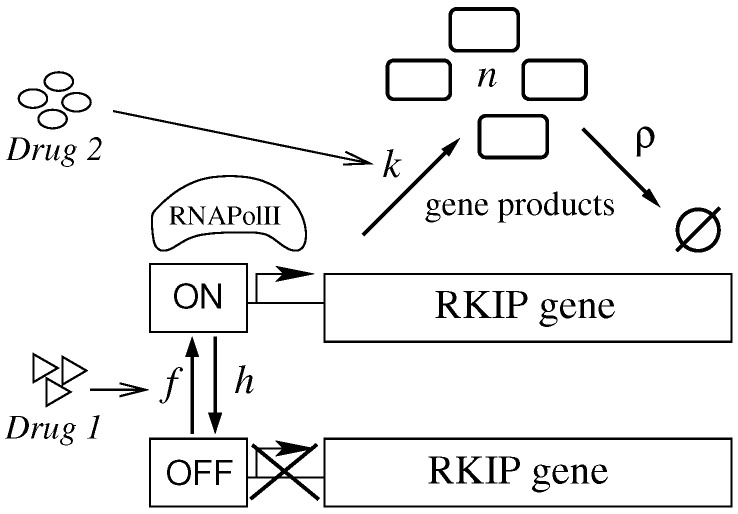
Drugs aiming at kinetic rates of RKIP transcription. RNAPolII binds to the promoter region (when it is ON) of RKIP gene to synthesize mRNA (gene products). The switching between ON and OFF states is dependent on the regulatory components (proteins) surrounding the gene. *Drug* 1 targets an activator protein and aims to increase the time of exposition of the promoter for binding of RNAPolII. *Drug* 2 affects only the promoter by increasing its efficiency or the affinity of the promoter to the RNAPolII. A treatment consists of administering a single or combination drugs following a dose agenda.

Our model for regulation of gene expression suggests the design of multiple treatment strategies aiming to increase expression of RKIP gene. The model gives four kinetic rates that we may target by drugs. In the specific case of the RKIP gene, one can attempt to increase *k* and *f* or to decrease ρ and *h*. Those rates can be affected individually or collectively, in a coordinated manner, in the case of a multi-drug therapy. In the scheme shown in Figure 1, we consider the particular cases in which the treatment aims to: (1) increase *f*; (2) increase *k*; and (3) increase *f* and *k* concomitantly. We assume that a given treatment targets a rate exclusively—that is, the non-targeted rates will remain constant during treatment. Two mechanisms can be used for increasing RKIP expression levels:

(1) Promoter demethylation. The downregulation of RKIP has been associated with promoter methylation in many cancer types [45,46,58,59,60]. We suppose the use of a demethylation agent, namely, 5-Azacytidine (5-AzaC), to revert that (see scheme given in Figure 2A in [22]). Consequently, the expression levels of RKIP would be increased as previously tested in the triple-negative breast cancer (TNBC) cell line SUM159 and esophageal cell lines TE-1 and TE-13 [48,58].

(2) Transcription factors regulation. The tumor environment is an inexhaustible source of signals that induce a change in the amounts of transcription factors regulating gene expression and, consequently, the phenotypes of tumor cells. One possible treatment is to use nitric oxide (NO) or NO donors acting as direct or chemosensitizer anti-cancer agents [61]. For example, NO [62] affects tumor growth by downregulating the functional quantities of NF- κ B and SNAIL, which, in reduced quantities, are associated with an increase in RKIP expression.

Thus, one may propose a therapeutic strategy using NO donors, such as (Z)-1-[2-(2-aminoethyl)-N-(2-ammonio-ethyl) amino] diazen-1-ium-1, 2-diolate (DETANONOate) [32,51,63,64], to maintain a constant inhibition of the NF- κ B/SNAIL loop. Indeed, RKIP was increased in treatments with DETANONOate designed for inhibiting the epithelial–mesenchymal transition (EMT) and invasion in metastatic human prostate carcinoma cell lines, which was corroborated in mice bearing tumor xenografts [51].

NO donors were used in combination with photosensitizers to increase the efficacy of the photodynamic therapy inhibiting proliferation of murine melanoma cells [65]. Note, however, that NO plays a dual role [66] since, in low-doses, it promotes carcinogenesis [67,68] Indeed, low levels of photodynamic therapy induce low levels of NO, which contributes to an anti-apoptotic response by NF- κ B/Snail/RKIP loop [69]. In this manuscript, we assume that the concentrations of NO within cancer cells are sufficiently large (micromolar levels [70]) to ensure its role in promoting an increase in the amount of RKIP transcripts (see scheme in Figure 2B in [22]).

The choice of 5-AzaC and DETANONOate was motivated because of a sufficiently good characterization of their mechanisms of action on signaling pathways that participate in upregulating RKIP expression. However, alternative treatment designs might be used to promote RKIP expression enabling a synergistic association with cancer therapies, such as Sorafenib associated with Gemcitibine [71] or Erlotinib [72] in lung cancers, and Gemcitibine with Sorafenib in pancreatic cancer [73]. Topoisomerase I inhibitor 9NC [74] and anti-mitotic agents ENMD-1198 and MKC-1 have also demonstrated the upregulating effects of RKIP [75].

However, finding drugs specifically targeting the promoter demethylation of RKIP gene or transcription factors regulating RKIP expression levels is a challenge beyond the scope of this manuscript. Therefore, as a strategy to introduce our methodology, to set clinically relevant timescales, and to show the difficulties of combining multiple drugs with multiple targets and timescales, we consider non-specific drugs. In that case, we are only considering the effect of the drug on the gene that we describe. The cellular level effects will not be considered here as those would require a more complex approach in which the dynamics of the expression levels of multiple potentially interacting genes would need to be considered.

### 2.4. An Approach for Investigating Treatment Effects on RKIP Expression Dynamics

Our treatment strategies aim to increase *f* and *k*. We assume that the drug effectiveness on those quantities decays exponentially and that the kinetic rates will return to their pre-treatment values. Hence, once treatment starts, the rates *f* and *k* become time-dependent with $f_{0}$ and $k_{0}$ being the OFF to ON and the synthesis rates, respectively, before treatment. We denote the fractional effect of a drug dose on the value of a kinetic constant by ξ , where 0<ξ≤1 . We assume that, at the maximum tolerated dose, the effect of the drug is given by ξ=1 and that this dose raises the targeted rates to $f_{1}$ and $k_{1}$ .

This does not imply the assumption of maximal efficiency of the drug effectiveness. Indeed, we consider that ξ is the net effect of a given dose on its targeted rate. Hence, a given dose smaller than the maximum tolerated one will instantaneously change its target rate to $\xi_{a}f_{1}$ and $\xi_{b}k_{1}$ , where $\xi_{a}$ and $\xi_{b}$ are non-linear functions of the dose that need to be formulated accordingly with experimental data. The time-dependent OFF to ON switching rate, f(t) , is

(5) $$f\left(t\right)=\left\{\begin{array}{cc} f_{0}, & 0\leq t<\tau_{1}\\ f_{0}+\left[f_{s}\left(\tau_{j}\right)-f_{0}\right]e^{-\lambda_{a}\left(t-\tau_{j}\right)},\; & \tau_{j}\leq t<\tau_{j+1} \end{array}\right.$$

and k(t)

(6) $$k\left(t\right)=\left\{\begin{array}{cc} k_{0}, & 0\leq t<\tau_{1}\\ k_{0}+\left[k_{s}\left(\tau_{j}\right)-k_{0}\right]e^{-\lambda_{b}\left(t-\tau_{j}\right)},\; & \tau_{j}\leq t<\tau_{j+1} \end{array}\right.$$

where j=1,…,J−1 denotes the *j*-th drug application, *J* is the amount of drug doses, and $\tau_{j}$ is the time of the *j*-th drug application. At each application of the drug, the steady state condition is that of the untreated system, namely $f_{0}$ and $k_{0}$ . $\left(\lambda_{a},\lambda_{b}\right)$ denote the rates of exponential decay of the effect of the drugs on the rates (f,k) .

As previously mentioned, we are considering that the effect of the drugs on their targeted rates is fast enough to be considered as instantaneous. Therefore, the values of the rates *f* and *k* immediately after the arrival of the *j*-th dose, respectively denoted by $f_{s}\left(\tau_{j}\right)$ and $k_{s}\left(\tau_{j}\right)$ , will be considered as the initial conditions during the interval $\tau_{j}\leq t<\tau_{j+1}$ . Note that the *j*-th dose adds up to the drug amounts that are remainders from previous applications. Hence, the initial condition at the time $\tau_{j}$ is written as:

(7) $$\begin{array}{ccc} f_{s}\left(\tau_{j}\right) & = & f_{1}\sum_{i=1}^{j}\xi_{a}\left(\tau_{i}\right)e^{-\lambda_{a}\left(\tau_{j}-\tau_{i}\right)}, \end{array}$$

(8) $$\begin{array}{ccc} k_{s}\left(\tau_{j}\right) & = & k_{1}\sum_{i=1}^{j}\xi_{b}\left(\tau_{i}\right)e^{-\lambda_{b}\left(\tau_{j}-\tau_{i}\right)}, \end{array}$$

where the dose may be calibrated to generate a differential effect at each instant to prevent toxic accumulation of drug quantities.

### 2.5. An Approximate Description of the Stochastic Binary Gene Expression Dynamics with Time-Dependent Kinetic Rates

The randomness of intracellular phenomena suggests a description of the treatment effects on expression of RKIP gene to be built in terms of a stochastic process. Figure 1 suggests the existence of two random variables that determine the state of the system: the gene state (being ON or OFF), and the number of gene products, denoted by *n*. The description of the dynamics of the state of the system is given in terms of a probability distribution

(9) $$\Pi (\alpha_{n}\left(t\right),\beta_{n}\left(t\right)),$$

where $\alpha_{n}\left(t\right)$ , or $\beta_{n}\left(t\right)$ , denote the probability of finding *n* proteins at time *t* when the gene is ON, or OFF, respectively. A master equation governing the probability distribution for an externally regulated gene can be written as:

(10) $$\begin{array}{ccc} \frac{d\alpha_{n}}{dt} & = & k\left(t\right)\left(\alpha_{n-1}-\alpha_{n}\right)+\rho \left[\left(n+1\right)\alpha_{n+1}-n\alpha_{n}\right]-h\alpha_{n}+f\left(t\right)\beta_{n}, \end{array}$$

(11) $$\begin{array}{ccc} \frac{d\beta_{n}}{dt} & = & \rho \left[\left(n+1\right)\beta_{n+1}-n\beta_{n}\right]+h\alpha_{n}-f\left(t\right)\beta_{n}, \end{array}$$

where *h* and ρ are constants and Equations (5) and (6) give f(t) and k(t) . The existence of time-dependent coefficients is difficult to solve Equations (10) and (11) analytically, and some numerical methods need to be employed. The interpretation of the master equation is built in terms of the coefficients k(t) , ρ , f(t) , and *h* as presented on the description of the Equations (1)–(4) and the cartoon shown on Figure 1.

Here, we propose to approximate the dynamics of the kinetic rates f(t) and k(t) of Equations (5) and (6) as piece-wise functions assuming constant values during sufficiently short time intervals ensuring that the difference between the exact and approximated values lies within a given error size (see Equation (A1) in the Appendix A.1). Then, we may consider the model for constant kinetic rates during an interval that is exactly solvable. In that case, the initial condition of an interval is the final condition on its previous neighbor.

### 2.6. An Exactly Solvable Model for Benchmarking Cancer Treatment Aiming to Modulate Gene Expression Levels

The master Equations (10) and (11) with constant coefficients has been already proposed, and it is fully solvable at the stationary [20] and time-dependent regimes [21]. The existence of exact solutions enables one to calculate the time-dependent functions governing the first and the second moment of the number of gene products [22]. Here, we write the explicit expressions governing the dynamics of the average number of gene products, 〈n〉(t) and the standard deviation, $\sigma \left(t\right)=\sqrt{\langle n^{2}\rangle \left(t\right)-{\langle n\rangle }^{2}\left(t\right)}$ . We use Equations (1)–(4) and define the following constants:

(12) $$N=\frac{k}{\rho };\;\;\;A_{s}=\frac{f}{f+h};\;\;\;\epsilon =\frac{f+h}{\rho },$$

which are, respectively, the steady state expected number of gene products in the case of a gene being fully ON, (we call this the *maximal mRNA number* *N*); the steady state probability for the gene to be ON ( $A_{s}$ ); and the ratio of the gene switching rate between ON and OFF states to the degradation rate of the gene products (we call this the *switching speed* ϵ ).

The average number of mRNAs and the standard deviation at the steady state regime are, respectively, denoted by ${\langle n\rangle }_{s}$ and $\sigma_{s}$ . We write them as functions of the parameters of Equation (12):

(13) $$\begin{array}{ccc} {\langle n\rangle }_{s} & = & A_{s}N, \end{array}$$

(14) $$\begin{array}{ccc} \sigma_{s}^{2} & = & {\langle n\rangle }_{s}\left(1+N\frac{1-A_{s}}{1+\epsilon }\right). \end{array}$$

The dynamics of the average and standard deviation are denoted by 〈n〉(t) and σ(t) , respectively, and we write:

(15) $$\begin{array}{ccc} \langle n\rangle (t) & = & {\langle n\rangle }_{s}+Y\;e^{-\epsilon \rho t}+V\;e^{-\rho t}, \end{array}$$

(16) $$\begin{array}{ccc} \sigma^{2}\left(t\right) & = & \sigma_{s}^{2}+U_{1}\;e^{-\epsilon \rho t}+V\;e^{-\rho t}+W_{1}e^{-(1+\epsilon )\rho t}+X_{1}e^{-2\rho t}-Y^{2}e^{-2\;\epsilon \rho t}. \end{array}$$

The coefficients of the exponentials are integration constants given on the Appendix A.4. These solutions are obtained from a system of ordinary differential equation coupling the moments *A*, $\langle n_{\alpha }\rangle$ , and $\langle n^{2}\rangle$ , where the exact forms are given in Appendix A.5.

Equations (15) and (16) enable us to compute the evolution of the average number of products from the RKIP gene and its standard deviation using a piece-wise representation of the time-dependent rates f(t) and k(t) as we show in the next section.

The decaying rate to steady state of both 〈n〉(t) (Equation (15)) and σ(t) (Equation (16)) can be established in terms of ϵ . For ϵ≫1 , the terms $e^{-\epsilon \rho t}$ , $e^{-(\epsilon +1)\rho t}$ become null much faster than the term $e^{-\rho t}$ , which determines the approaching to steady state. Alternatively, for ϵ≪1 , the term $e^{-\rho t}$ will govern lifetime of the dynamical regime, that is, the regime during which 〈n〉(t) and σ(t) are varying with time.

Additionally, one may notice that ϵ also reflects the ratio of the gene switching frequency to the degradation rate, the characteristic times of the two processes being coupled. Equation (14) indicates that $\sigma_{s}^{2}\to {\langle n\rangle }_{s}$ when ϵ≫N>1 . This coincides with the decaying to steady state being determined by the gene product degradation rate, as it happens on a Poisson process. On the other hand, when the gene switching decaying rate to steady state is prevalent, for ϵ≪1 , $\sigma_{s}^{2}$ will have larger values. Then, the gene switching will have a stronger effect on the fluctuations of the number of gene products.

### 2.7. Parameter Values and Conditions for Treatment Simulations

The exactly solvable model for binary stochastic gene expression enables the simulation of the dynamics of the response to treatment measured in terms of both the average expression and random fluctuations around the mean of a hypothetical (abstract) gene. However, such a choice may turn hard the provision of insights for one willing to design a treatment to modulate the expression of a master regulating gene. One goal is the design of an optimal agenda of application of treatment to control both the average and standard deviations of gene products to remain within specific ranges.

The instants of application of each drug, denoted by τ , are strongly related to the characteristic times of the system composed of a gene and its interacting drugs. Among those characteristic times, the half-lives of the drugs, denoted by $\lambda_{i}$ where *i* labels a given drug, and degradation rate of the gene products, denoted by ρ , are widely used. However, as illustrated in a previous subsection, the introduction of the promoter switching provides an additional characteristic time, denoted by ϵρ , which will have very important effects on treatment response, as shown below. Hence, the choice of the system RKIP, 5-AzaC, and DETANONOate helps us to set a few biologically reasonable parameter values to perform our analysis. Adapting our framework for different systems, however, is a fully feasible task.

Here, the rates related to DETANONOate and 5-AzaC are, respectively, denoted by an index *a* and *b*. Hence, the degradation rates of DETANONOate (acting on *f*) and 5-AzaC (acting on *k*) are denoted by $\lambda_{a}$ and $\lambda_{b}$ , respectively. Their values are $\lambda_{a}=0.05\;h^{-1}$ [76] and $\lambda_{b}=0.25\;h^{-1}$ (DB00928 entry at DrugBank [77]).

A separate challenge for designing a therapy targeting a specific gene is the formulation of an effective model for the effect of treatment on a specific gene parameter. As this is beyond the scope of the current study, we defined a quantity denoted by ξ , which will indicate the effect of a given treatment dose on its target parameter. For the maximum tolerated dose, the effect will be considered as maximal and indicated by ξ=1 . When we have 0<ξ<1 , we are indicating that the dosage is smaller than maximal. Note, however, that we do not have a mathematical model relating ξ to the drug dosage, and its construction requires specific experiments.

We denote the response generated on the kinetic rates by treatment at maximum tolerated dose by $\xi_{a}=\xi_{b}=1$ . For a single dose, we assume that the drugs change the values of their targeted rates instantaneously after application, and for maximum tolerated dose, $f\to f_{1}$ and $k\to k_{1}$ , as indicated by Equations (7) and (8), respectively.

The specific values of dosage of both drugs considered here have been set in previous studies. The dose values and effects on RKIP expression levels may vary by up to one order of magnitude. For example, experimental analysis of human prostate metastatic cells (DU145 and PC-3) treated with 1000 μM DETANONOate showed upregulation of RKIP mRNA for 4 and 12 h post-treatment [51]. Triple-negative breast cancer cells (SUM159) also increase in RKIP mRNA 1.4 fold when treated with 500–2000 μM 5-AzaC for 72 h [48], and human esophageal cancer cell (TE-1 and TE-13 cells) treated with 2 μ g/mL 5-AzaC showed a 1.5 –10-fold increase in RKIP expression [58].

**Figure 2:**
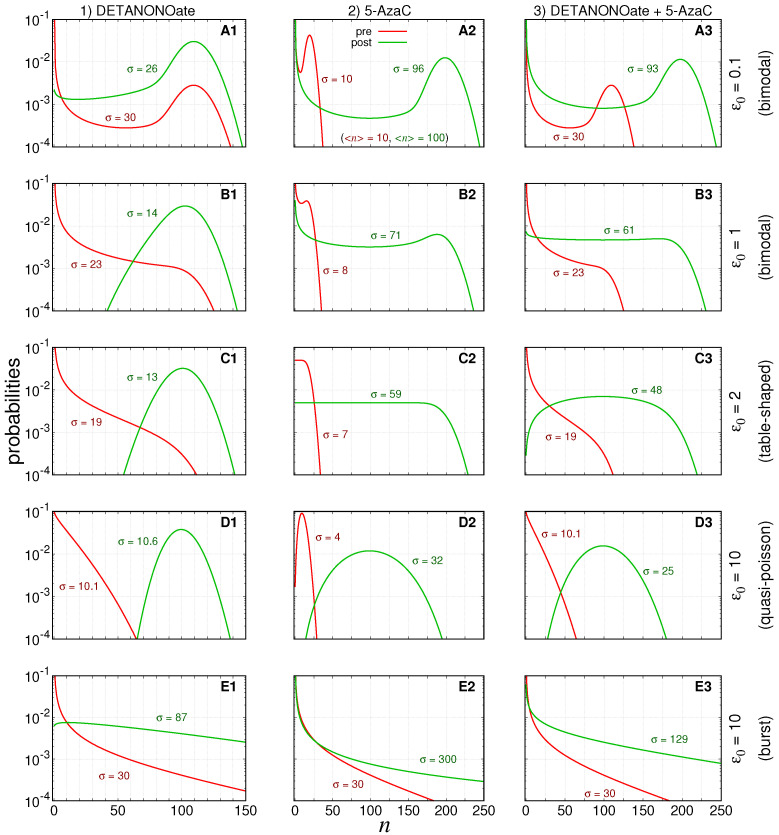
Probability distributions. The red lines indicate pre-treatment probabilities, while the green lines indicate the aimed probabilities governing the post-treatment number of gene products. The post-treatment distribution parameters were set aiming to increase the average number of transcripts to be ≈100. We disregarded the effect of the new parameter values on the variances, i.e., the heterogeneity of treatment response. We consider five initial conditions for the gene switching speed, $\epsilon_{0}=\left(0.1,1,2,10,10\right)$ , arranged in rows and indicated by (**A1**–**A3**), (**B1**–**B3**), (**C1**–**C3**), (**D1**–**D3**) and (**E1**–**E3**) in the labels of respective graphs. (**D**,**E**) have the same value for $\epsilon_{0}$ but represent different because of the differing values of $A_{0}$ . The distributions for the treatment designs are arranged in columns: (1) DETANONOate aiming the *f* rate, (2) 5-AzaC aiming the *k* rate, and (3) both drugs aiming both *f*, and *k* rates, simultaneously.

The treatment induces a change in the kinetic parameters of the model. As a consequence, all quantities depending on those parameters will be changed. Hence, $f_{0}\to f_{1}$ and $k_{0}\to k_{1}$ causes a change of $\epsilon_{0}\to \epsilon_{1}$ , and a change on the steady state values of the statistical quantities ${\langle n\rangle }_{s,0}\to {\langle n\rangle }_{s,1}$ , and $\sigma_{s,0}\to \sigma_{s,1}$ accordingly with Equations (12)–(14). The expected steady state probability distributions governing the number of RNA transcripts instantaneously after treatment application as shown in Figure 2. However, because the drug effects decay exponentially with a rate determined by their half-life, the system does not reach those new steady state values and tends to return to the pre-treatment conditions instead.

Here, we assume that the gene product of RKIP are mRNAs, whose degradation rate is $\rho =0.17\;h^{-1}$ [78]. The pre-treatment condition, occurring for $0\leq t<\tau_{1}$ , is not shown because we assume it as a stationary state, and thus we set $\tau_{1}=0$ . The pre-treatment condition is characterized by a low average copy number of RKIP mRNA’s (here, chosen as ${\langle n\rangle }_{0}=10$ ). We arbitrarily assume that, in pre-treatment conditions, the levels of mRNAs are eight-times below what would be expected to be found in a non-metastatic cell. Hence, the treatment is assumed to be successful if it drives the probability of finding less than ${\langle n\rangle }_{T}\approx 80$ mRNAs to a negligible value. This is an important requirement to minimize heterogeneity in a treatment response.

The randomness of intracellular processes causes a variability in the treatment response even under the hypothetical conditions in which all individuals of a population of genetically identical cells absorb the same drug dosage. The heterogeneity of the response can be quantified by the standard deviation of the number of gene products. Hence, we first set ${\langle n\rangle }_{1}=100$ as the expression level aimed by treatment. This value is chosen assuming that a gene expressing an average of 100 mRNAs in a Poisson regime has the threshold value $80={\langle n\rangle }_{1}-2\sigma_{1}$ . Here, $\sigma_{1}=\sqrt{{\langle n\rangle }_{1}}$ is the standard deviation of a Poisson distribution with ${\langle n\rangle }_{1}$ as its average.

The dynamics of *f* and *k* are described by Equations (5) and (6). We approximate them as piece-wise functions and compute the error using integrals of the exponential decays along each subinterval. Then, we set the length of each subinterval by fixing its error. The piece-wise approximation enables the use of 〈n〉(t) (Equation (15)) and σ(t) (Equation (16)) to describe the expression of RKIP.

**Figure 3:**
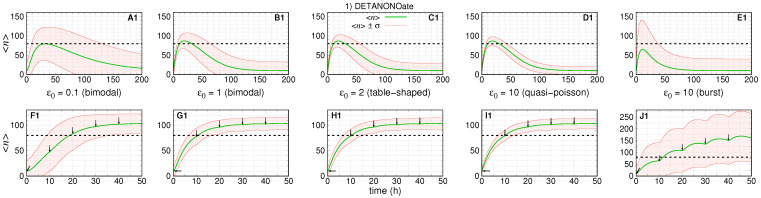
The dynamics of the number of RKIP mRNA (green solid line) and their standard deviation (red solid line) along the time for treatment aiming *f* kinetic rate. The black dashed line at 80 indicates an arbitrary threshold on the number of RKIP mRNA. (**A1**–**E1**) show single doses of drug with different progressive values to $\epsilon_{0}$ (shown within the respective graph). (**F1**–**J1**) show the same initial conditions that (**A1**–**E1**) for 5 doses with time interval of 10h (indicated by arrows). All doses are in maximum tolerance ( $\xi_{a}=1$ ).

**Figure 4:**
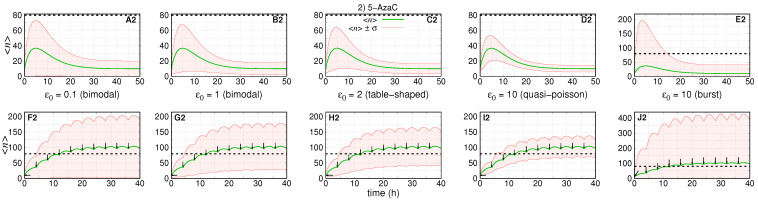
The dynamics of the number of RKIP mRNA (green solid line) and their standard deviation (red solid line) along the time for treatment aiming *k* kinetic rate. The black dashed line at 80 indicates an arbitrary threshold on the number of RKIP mRNA. (**A2**–**E2**) show single doses of drugs with different progressive values to $\epsilon_{0}$ (shown within the respective graph). (**F2**–**J2**) show the same initial conditions that (**A2**–**E2**) for 10 doses with time interval of 4h (indicated by arrows). All doses are in maximum tolerance ( $\xi_{b}=1$ ).

**Figure 5:**
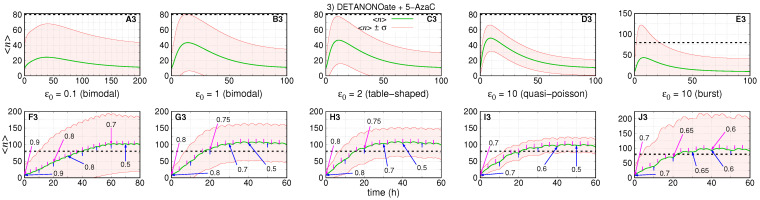
The dynamics of the number of RKIP mRNA along the time for treatment with two drugs that aim at *f* and *k* kinetic rates are shown in green solid lines, and their standard deviations are shown in red solid lines. The threshold for RKIP is 80, and it is shown within graphs as a black dashed line. (**A3**–**E3**) show the single doses of both drugs for different progressive values to $\epsilon_{0}$ (indicated within each graph). (**F3**–**J3**) show the same initial conditions that (**A3**–**E3**) for multiple fractional doses of drug that target *f* (or *k*) with time interval of 10 h (or 4 h), in order, with 8 (or 20) doses in (**F3**) and 6 (or 15) doses in (**G3**–**J3**). Arrows indicate the agenda, with time moments and fractions ( $\xi_{a}$ and $\xi_{b}$ ) for each drug, DETANONOate (aiming *f*) in blue and 5-AzaC (aiming *k*) in magenta. The numbers on the larger arrows indicate the fraction of the dose applied at that time and those that follow (arrows without numbers). The agenda is also shown in Table 1.

A single dose will not be sufficient for keeping ${\langle n\rangle }_{1}\approx 100$ because of the exponential decay of the drug effect on the kinetic constants (as will be shown on graphs A–E of Figure 3, Figure 4 and Figure 5). Hence, multiple doses are necessary, and we determine the intervals between applications of DETANONOate and 5-AzaC as, respectively, 10h and 4h . These numbers were chosen to ensure that 〈n〉(t)≈100 during a sufficiently long time interval, and their choice is based on the degradation rates of each drug.

The treatment changes the dynamical properties of the gene switching. *When we consider DETANONOate*, the pre-treatment probability of finding the gene ON is $A_{0}=\frac{f_{0}}{f_{0}+h}$ . The drug dose delivered at $\tau_{j}$ causes $f_{0}\to f_{s}\left(\tau_{j}\right)$ instantaneously as defined at Equation (7). Then, the aimed steady state probability for the gene to be ON is $A_{1}=\frac{f_{s}\left(\tau_{j}\right)}{f_{s}\left(\tau_{j}\right)+h}$ . The pre (and instantaneously post) treatment values of the gene switching frequency are, respectively, $\epsilon_{0}=\frac{f_{0}+h}{\rho }$ and $\epsilon_{1}=\frac{f_{s}\left(\tau_{j}\right)+h}{\rho }$ . *When we consider 5-AzaC* the probability of finding the gene ON, given by $A_{0}=\frac{f_{0}}{f_{0}+h}$ , and gene switching frequency $\epsilon_{0}=\frac{f_{0}+h}{\rho }$ , will both remain constant, while the value of $k_{0}\to k_{s}\left(\tau_{j}\right)$ instantaneously after drug application at $\tau_{j}$ —see Equation (8)—such that $N_{0}=\frac{k}{\rho }\to N_{1}=\frac{k_{s}\left(\tau_{j}\right)}{\rho }$ .

As we are using an exactly solvable stochastic model, we can map its qualitative features in terms of the relations between its kinetic parameters. For example, the value of ϵ in Equation (12) is a key parameter determining the shape of the steady state probability distributions shown in Figure 2. Hence, the interpretation of our results will remain useful in the analysis of specific experimental designs despite the need for specific values for the parameters of the model.

Hence, our choice for both pre- and post-treatment average values of mRNAs are arbitrary and selected for the clearness of presentation of our results and predictions. A four- to eight-fold increase in the median of mRNA numbers has been reported in PCPG (pheochromocytoma and paraganglioma), CHOL (cholangiocarcinoma), and SARC (sarcoma) as shown in [32], Figure 1. The RKIP expression levels in LIHC (liver hepatocellular carcinoma) [79], GBMLGG (glioma) [80] and STES (stomach and esophageal carcinoma) [81] were increased by up to two times.

Other comparisons of RKIP expression levels in breast cancer (BRCA and TNBC) [82,83], skin cancer (SKCM) [84,85], and colorectal cancers (COADREAD) [57,86,87] indicated a negligible change on the median. In all aforementioned cancers, the variability in the numbers of transcripts of RKIP was larger in tumor cells than in normal cells. One advantage of the use of a stochastic model is the possibility of raising possible explanations for such features.

## 3. Results

We simulate three treatment scenarios: (1) denotes the effect of maximum tolerated dose of DETANONOate; (2) denotes the effect of maximum tolerated dose of 5-AzaC; (3) denotes the effect of fractional doses of the two drugs together. The treatment response is quantified in terms of the time for the average value to reach the threshold and the standard deviation. These two quantities are strongly dependent on the values of $\epsilon_{0}$ , as we will show in the next subsections. The values of ρ , $\lambda_{a}$ , and $\lambda_{b}$ are assumed to remain constant during treatment. All rates (*k*, *f*, and *h*) are given in $h^{-1}$ .

The values of the parameters were selected for simulating treatment conditions whose probability distributions governing the expression of pre-treated cells indicate qualitatively distinguishable steady state regimens as recently classified [23]. Figure 2 shows pre-treatment (and aimed post-treatment) steady state probability distributions governing the numbers of RKIP mRNAs in red (and green).

The values of the parameters in each row are set to ensure that graphs A indicate bimodal distributions, graphs B are distributions close to the limit of the bimodal regime, graphs C indicate the regime in which the probabilities are table-shaped for $A_{0}=A_{1}=0.5$ (as indicated in graph C2), graphs D indicate the quasi-Poisson distributions, and graphs E denote the bursting limit. The curves of the steady state probability distributions of finding *n* mRNAs, within the Figure 2, are computed by Equation (A33) and is denoted by ${\tilde{\phi }}_{n}$ in the Appendix A.6.

The parameters of the pre- and post-treatment probability distributions are fixed such that the steady state average number of mRNA’s will be ∼10 and ∼100, respectively. The bimodal distributions indicate that the mRNAs synthesized while the gene is ON will degrade quickly after switching to the OFF state. For the table-shaped and quasi-Poissonian limits, the switching between ON–OFF states are, respectively, slow and fast enough to ensure that the mRNA degradation is compensated by its synthesis to generate the specific distributions. The burst limit is characterized by very short ON states during, which the synthesis is very efficient, while the OFF state duration is proportionally very long.

In next subsections, we present the results of simulations of the treatment response dynamics considering the five pre-treatment conditions shown in Figure 2. The trajectories of 〈n〉(t) and σ(t) were obtained, respectively, using Equations (15) and (16) within the discrete intervals used to approximate the kinetic rates after treatment injection given by Equations (7) and (8).

Figure 3, Figure 4 and Figure 5 show the response after application of a single (or multiple) doses on graphs labeled as A–E (or F–J) followed by the number indicating the treatment scenario (1, 2, or 3). The pre-treatment conditions have $\epsilon_{0}=\left(0.1,1,2,10,10\right)$ . The black dashed lines indicate the aimed average value after treatment. The green lines indicate 〈n〉(t) , and the red lines indicate the values of 〈n〉(t)±σ(t) . The parameters of the treatment are set to enable the average number of gene products to reach the post-treatment regime shown in Figure 2 for each set of parameter values.

### 3.1. Treatment Aiming at the OFF to ON Gene State Switching Rate

Figure 3 shows a simulation of the dynamics of the average number of mRNAs and its standard deviation resulting from a change on *f* after introduction of DETANONOate. The absolute error of each subinterval of the piece-wise approximation for f(t) is $1\times 10^{-4}$ . We assume the rates (k,h,ρ) are constant during treatment. For obtaining the Figure 3 A1–D1,F1–I1, we set $\left(k_{0},N_{0}\right)=\left(k_{1},N_{1}\right)=\left(18.5,\;110\right)$ and $\left(A_{0},A_{1}\right)=\left(0.09,\;0.9\right)$ . Figure 3E1,J1 have $\left(k_{0},N_{0}\right)=\left(k_{1},N_{1}\right)=\left(166.7,\;1000\right)$ and $\left(A_{0},A_{1}\right)=\left(0.01,\;0.1\right)$ to ensure the bursting gene expression regime during the pre-treatment stage.

On Figure 3A1–E1 (or Figure 3 F1–J1), we set $f_{0}=\left(0.0015,\;0.015,\;0.03,\;0.15,\;0.017\right)$ and $f_{1}=\left(0.14,\;1.37,\;2.73,\;13.65,\;0.18\right)$ , such that, h=(0.015,0.15,0.3,1.52,1.65) and $\epsilon_{1}=\left(0.9,\;9.1,\;18.2,\;91,\;11\right)$ with the given values of $A_{0}$ , $A_{1}$ and ρ . The maximum tolerated dose is considered to cause the steady state ON state probability to be multiplied by ten, as indicated by the values of $A_{0}$ and $A_{1}$ .

The first and second rows of graphs of Figure 3 show the simulation of the dynamics of expression after one and five drug doses, respectively, under five pre-treatment conditions.

Figure 3A1,B1 show the dynamics of response of the slow switching gene, a regime of synthesis of mRNAs whose numbers are governed by a bimodal distribution at the steady state. For $\epsilon_{0}=0.1$ , we have the slowest treatment response and return to pre-treatment conditions.

The average number of mRNAs reaches a maximum of ∼80 after ∼30 h. The standard deviation is the second largest. Figure 3A1–D1 show the average mRNA number reaching or crossing the threshold in our simulations, the time for the average number to reach a maximum when $\epsilon_{0}\geq 1$ is ∼20 h, and the noise of the response decreases as we increase $\epsilon_{0}$ . The exception is shown on Figure 3E1 where the noise is the largest and the time for the average number to reach a maximum is ∼10 h. In this case, the average number does not cross the threshold, and the lower line 〈n〉(t)−σ(t) does not appear.

The response to multi-dose treatment is shown in Figure 3F1–J1. The interval between doses was chosen to enable a sigmoidal-like response characterizing two distinct levels of expression with the average number increasing from 10 to at least ∼100. Figure 3F1 shows that the slow switching gene also causes the slowest response as 〈n〉(t) crosses the threshold after ∼18 h. The curve for 〈n〉(t)−σ(t) also crosses the threshold after ∼35 h, which ensures a less-heterogeneous response to treatment. Figure 3G1–I1 show simulations for increasing the values of gene switching.

Those simulations show that 〈n〉(t) crosses the threshold after ∼10 h, and the curves 〈n〉(t)−σ(t) reach higher values, which establishes the response to treatment with the minimal heterogeneity. Figure 3J1 shows the response of a burst gene, which 〈n〉(t) crosses the threshold after ∼12 h and reaches a maximal value of ∼170. However, this regime causes the noisiest response as indicated by 〈n〉(t)−σ(t) not crossing the threshold. Note also that there are some “bumps” as the effect of the single dose is close to the maximum by the time of the next drug application. It is possible to prevent the bumps; however, we decided to show them to demonstrate that the time interval between drug dosages also requires one to consider the dynamics of the targeted gene.

### 3.2. Treatment Aiming at the RKIP mRNA Synthesis Rate

Figure 4 shows the dynamics of the average number of RKIP mRNAs and the standard deviations under treatment with 5-AzaC. The absolute error of each subinterval of the piece-wise approximation for k(t) is $1\times 10^{-4}$ . We assume that the rates (f,h,ρ) remain constant during treatment, which implies $A_{0}=A_{1}$ and $\epsilon_{0}=\epsilon_{1}$ also remain constant. For Figure 4A2–D2,F2–I2, we set $\left(k_{0},N_{0},\;k_{1},N_{1}\right)=\left(3.3,\;20,\;33,\;200\right)$ , $A_{0}=0.5$ and f=h=(0.008,0.08,0.17,0.83) . For Figure 4E2,J2, we set $\left(k_{0},N_{0},\;k_{1},N_{1}\right)=\left(166.7,\;1000,\;1667,10,000\right)$ , $A_{0}=0.01$ and (f,h)=(0.017,1.65) .

The first and second rows of graphs of Figure 4 show the simulation of the dynamics of expression after one and ten drug doses, respectively, under five pre-treatment conditions.

The treatments do not affect either ϵ or ρ , the decaying rates of the system. Then, Figure 4A2–E2 show that the average number of mRNAs reach the maximum (∼35 molecules) after similar intervals of ∼5 h and reach pre-treatment conditions after ∼40 h. Figure 4A2 shows the condition with the second largest standard deviation. Figure 4A2–D2 show that the noise of the response decreases with the increase of $\epsilon_{0}$ . The burst regime shown in Figure 4E2 leads to the noisiest response. Indeed, 〈n〉(t)−σ(t) remains negative, while 〈n〉(t)+σ(t) exceeds the threshold more than 2× .

The response to multi-doses treatment is shown in Figure 4F2–J2. The interval between doses was chosen to enable a sigmoidal-like response with the average number reaching at least 100. All graphs show that 〈n〉(t) crosses the threshold after ∼10 h . This is because the response to treatments and interval between drug application is the same in all scenarios. Since both $\epsilon_{0}$ and ρ are not affected, the decaying rates to pre-treatment conditions are not affected.

In all scenarios, the curves for 〈n〉(t)−σ(t) do not cross the threshold because the treatment does not increase $\epsilon_{0}$ that would cause a reduction in the noise in mRNA synthesis in a given scenario. However, Figure 4F2–I2 show that 〈n〉(t)−σ(t) reaches higher maximum values as $\epsilon_{0}$ increases. Figure 4J2 shows the response in the case of transcriptional bursts, which leads to the most heterogeneous response to treatment as indicated by the curve 〈n〉(t)+σ(t) reaching the highest maximum.

### 3.3. Treatment with the Two DRUGS Concomitantly

Figure 5 shows the dynamics of the average number of mRNAs and its standard deviation under treatment with both drugs: DETANONOate and 5-AzaC. The absolute errors of each subinterval of the piece-wise approximation for f(t) and k(t) are $1\times 10^{-4}$ for both. The rates (h,ρ) remain constant during treatment. For Figure 5A3–D3 (or Figure 5F3–I3), we set $\left(k_{0},N_{0},\;k_{1},N_{1}\right)=\left(18.5,\;110,\;33,\;200\right)$ , $\left(A_{0},A_{1}\right)=\left(0.09,\;0.5\right)$ , $f_{0}=\left(0.0015,\;0.015,\;0.03,\;0.15\right)$ and $f_{1}=h=\left(0.015,\;0.15,\;0.3,\;1.5\right)$ . For Figure 5E3,J3, we set $\left(k_{0},N_{0},\;k_{1},N_{1}\right)$ = (167,1000,333,2000) , $\left(A_{0},A_{1}\right)=\left(0.01,\;0.05\right)$ , $f_{0}=0.017$ , $f_{1}=0.087$ and h=1.65 . The values of $A_{0}$ , $A_{1}$ and ρ results in $\epsilon_{1}=\left(0.18,\;1.8,\;3.6,\;18,\;10.4\right)$ on Figure 5A3–E3 (or Figure 5F3–J3).

Figure 5 shows the simulations of the dynamics of response to treatment. Graphs of the first row indicate the results of simultaneous application of a single dose of both drugs. The second row shows the results of applications of multiple doses with drug targeting *f* (or *k*) being applied every 10 h (or 4 h) according to the agenda in Table 1.

Figure 5A3 shows the dynamics of the treatment response when the initial conditions are set for a slow switching gene. The average number reaches a maximum of ∼25 at t∼ 35 h. The maximum of 〈n〉(t)+σ(t) is ∼70, a highly heterogeneous response if we consider that the maximum standard deviation is equal to the maximum average—that is, a super-Poissonian regime of gene transcription. The return to pre-treatment is slow as indicated by the decay of the average after ∼200 h.

In Figure 5B3–D3, the maximum average numbers of mRNAs are ∼40, ∼45, and 50, respectively, reached after 15, 12, and 8 h. The curves 〈n〉(t)−σ(t) and 〈n〉(t)+σ(t) become closer with the increase of $\epsilon_{0}$ as shown from Figure 5A3–D3. The burst limit is different and, despite the high value of $\epsilon_{0}$ , has larger noise as shown in Figure 5E3. At this limit, 〈n〉(t) maximum is ∼45 reached after ∼8 h. 〈n〉(t)+σ(t) reaches a maximum of ∼120, indicating the noisiest response. For all pre-treatment conditions, the average number does not cross the threshold.

The response to multi-doses treatment is shown in Figure 5F3–J3. Table 1 shows the sequences of applications of a given drug in fractions of maximal tolerated dose, $\xi_{a}$ and $\xi_{b}$ . The values were chosen aiming that the total amount of drugs is reduced in comparison with the single drug treatment. We also attempt to ensure a sigmoidal-like response such that the average number will reach at least ∼100. Figure 5F3 shows the dynamics of response for 8 (or 20) doses of drug targeting *f* (or *k*) with $\xi_{a}$ and $\xi_{b}$ ranging from 0.9 to 0.5 (details in Table 1).

The agenda enables a reduction of 20% in comparison with application of full doses ( $\xi_{a}=\xi_{b}=1$ ). As the pre-treatment gene is in a slow switching regime that leads to the slowest response as 〈n〉(t) crosses the threshold after ∼40 h. It also has a high noise as can be noticed by the values of 〈n〉(t)±σ(t) . Figure 5G–J3 show the simulations of the response for 6 (or 15) doses of drugs *a* (or *b*) with $\xi_{a}$ and $\xi_{b}$ ranging from 0.8 to 0.5 (see the Table 1).

The cumulative reduction in full doses range from 29% to 35% (or from 23% to 35% ) in $\xi_{a}$ (or $\xi_{b}$ ). Figure 5G3–I3 show that 〈n〉(t) crosses the threshold after ∼15 h. The curves 〈n〉(t)±σ(t) become closer as the value of ϵ increases, which indicates the direction to design treatment strategies with reduced response heterogeneity. Figure 5J3 shows the burst pre-treatment condition. Here, 〈n〉(t) crosses the threshold after ∼22 h and 〈n〉(t)+σ(t) reaches ∼200, which indicates the noisiest response.

### 3.4. Treatment Response Mapping $\xi_{a}$ and $\xi_{b}$ Fractional Effect of Drug Reduction

**Figure 6:**
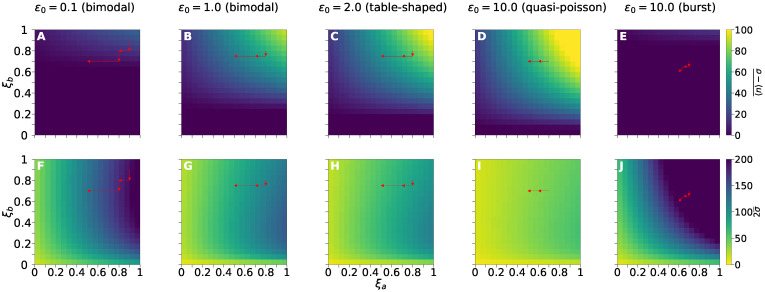
Effectiveness of treatment agendas measured by the average values $\bar{\langle n\rangle -\sigma }$ (top row) and $2\bar{\sigma }$ (bottom row) during the interval from 60 to 80 h after application of the first dose is presented. The vertical bars on the right give color code denoting the average values. In each square of a heatmap, we indicate the value of $\bar{\langle n\rangle -\sigma }$ (top row) and $2\bar{\sigma }$ (bottom row) as a result of the fractional dose effect $\xi_{a}$ and $\xi_{b}$ on rates *f* and *k*, respectively. The analysis was performed for the five pre-treatment conditions identified by the switching speed value $\epsilon_{0}$ labeling each column. For each pair ( $\xi_{a}$ , $\xi_{b}$ ), we simulated the application of DETANONOate (and 5-AzaC) every 10 h (and 4 h). For a treatment aiming RKIP gene, dose reduction to minimize cytotoxic effects for each $\epsilon_{0}$ consists on keeping $\left(\xi_{a},\;\xi_{b}\right)$ within regions with $\bar{\langle n\rangle -\sigma }$ above Graphs (**A**–**E**) and $2\bar{\sigma }$ below Graphs (**F**–**J**) specific thresholds. The yellow regions of the heatmaps indicate the more desirable domains evaluated in terms of $\bar{\langle n\rangle -\sigma }$ and $2\bar{\sigma }$ . The sequence of red arrows in each graph corresponds to the effect of dose fraction combinations in the agendas in Figure 5 and Table 1, for each pre-treatment condition.

Figure 6 shows the treatment response measured in terms of the average (not weighted) values $\bar{\langle n\rangle -\sigma }$ (Figure 6A–E), and $2\bar{\sigma }$ (Figure 6F–J) from 60 to 80 h after the first dose. Five pre-treatment regimes labeled by $\epsilon_{0}$ values are considered. The fraction of DETANONOate and 5-AzaC effect on *f* and *k* is indicated by $\xi_{a}$ and $\xi_{b}$ , respectively. We compute the average value of the function x(t) between instants $t_{i}$ and $t_{f}$ , denoted by $\bar{x(t)}$ , using the integral $\bar{x(t)}=\frac{1}{t_{f}-t_{i}}\int_{t_{i}}^{t_{f}}x\left(t\right)\;dt$ .

The absolute error of the piece-wise approximation for the exponential decay of the drug effect is set to $1\times 10^{-4}$ . To obtain each point of the heatmaps in Figure 6, we computed the dynamics of the treatment response to an agenda with doses with constant fractions $\xi_{a}$ and $\xi_{b}$ . DETANONOate was applied in eight doses with a time interval of 10 h, and 5-AzaC in 20 doses with an interval of 4 h. The grid is constructed by computing the trajectories for $\xi_{a}$ and $\xi_{b}$ varying from 0 to 1 by 0.05 increments.

Reduction of fractions of effects of dosage $\xi_{a}$ and $\xi_{b}$ for agendas shown in Table 1 and Figure 5 were based on heatmaps in Figure 6. The best scenario is located in the yellowest region of the heatmaps. The changes on the fractional effect of reduced doses over time are indicated by the sequence of red arrows within the graphs indicating each pre-treatment regime. Bimodal (Figure 6A,F) and burst (Figure 6E,J) regimes are the hardest pre-treatment conditions for dose reduction because there are almost no $(\xi_{a}$ , $\xi_{b})\to \left(0,\;0\right)$ that ensure sufficiently high values of $\bar{\langle n\rangle -\sigma }$ (Figure 6A,E) and low $2\bar{\sigma }$ (Figure 6F,J).

The additional pre-treatment conditions, namely the remaining bimodal, the table-shaped, and the quasi-poisson (Figure 6B–D,G–I), enable better scenarios for dose reduction because of the larger values of $\epsilon_{0}$ . Overall, all pre-treatment regimens enable a larger reduction of $\xi_{a}$ than of $\xi_{b}$ under the agenda proposed in Table 1 (see red arrows in Figure 6), because of the action of 5-AzaC on *k* (given in terms of $\xi_{b}$ ). Larger values of $\xi_{b}$ enable larger increases on average RKIP mRNA levels.

However, bimodal and burst regimens enable one to further reduce $\xi_{b}$ as the standard deviation remains constant or diminished, while the average RKIP mRNA numbers remain almost constant. However, though it is possible to reduce the variance, the average values do not reach sufficiently large values. Hence, we need to understand how treatment design may target the other kinetic rates, *h* and ρ , to enable an effective response independent of pre-treatment conditions.

### 3.5. Enhancing Ineffective Treatments Aiming at All Kinetic Rates

**Figure 7:**
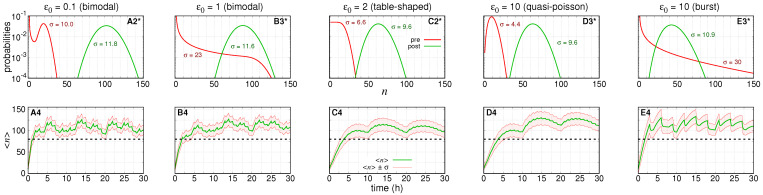
The enhanced treatment designs resulting from distributions of Figure 2A2,B3,C2,D3,E3. We simulate a hypothetical cocktail of drugs changing all rates (f,k,h,ρ) . The first row of graphs shows the pre- (red lines) and post-(green lines) treatment probability distributions governing the mRNA numbers. An asterisk is added to the labels to indicate that we are simulating the enhanced treatment design. The average number 〈n〉 for all pre-treatment distributions is 10 and for each post-treatment distribution is (103,88,63,63,44) following graphs from left to right, respectively. The second row shows the respective dynamic of responses to enhanced treatment design in (**A4**–**E4**). Green lines indicate the average numbers of RKIP mRNAs, 〈n〉 . Red lines show the one standard deviation around the average, 〈n〉±σ . Black dashed lines at 〈n〉=80 indicate the threshold separating the mRNA expression levels in a healthy (above line) and cancer (below) cell. The parameters of post-treatment distributions and the agenda of doses of each enhanced treatment design are shown in Appendix B.

Response to enhanced treatment is shown in Figure 7 for five qualitatively different initial conditions aiming at optimal distribution. The hypothetical drug cocktail targets all rates of the model and we reach an optimal response time and heterogeneity reduction for all qualitatively distinguishable initial conditions, which, in previous subsections, led to unsatisfactory results. The pre-treatment distributions are shown by the red curves within the graphs in Figure 2: A2, B3, C2, D3, E3, which, respectively, describe a bimodal, bimodal limit, table shaped, quasi-Poissonian, and burst steady state regimes of gene transcription.

Our goal was to keep the average numbers of mRNAs around 100 and their fluctuations above the threshold. The decaying rates of the drugs affecting the drugs *k*, ρ , *f*, and *h* are denoted by $\lambda_{i}$ , where *i* indicates the rate targeted by the drug. The decaying rate of the drugs is fixed in $h^{-1}$ : $\left(\lambda_{k},\;\lambda_{\rho },\;\lambda_{f},\;\lambda_{h}\right)=\left(0.25,\;6,\;0.05,\;0.053\right)$ , and the maximal tolerated dose of drug *i* is denoted by $\xi_{i}=1$ .

The values of $\lambda_{f}$ and $\lambda_{k}$ are the same used previously from DETANONOate and 5-AzaC, respectively, while $\lambda_{\rho }$ and $\lambda_{h}$ values were set based on the mRNA–microRNA binding duration time [88] and *h* in BACH1 half-life [89]. The time-dependence of h(t) and ρ(t) is described following the same framework used for k(t) and h(t) —see Equations (5)–(8). To obtain the dynamics of the average number of mRNAs and of the standard deviation, we extend the previous formulation of the master equation of Equations (10) and (11) to all kinetic rates.

The standard deviation of the post-treatment probability distribution is set to be

(17) $$\sigma_{1}=\sqrt{N_{1}A_{1}\left[1+N_{1}\frac{\left(1-A_{1}\right)}{1+\epsilon_{1}}\right]},$$

which has a local minimum at $N_{1}\to 0$ and a local maximum at

(18) $$A_{1}=\frac{1}{2}\left(1+\frac{1+\epsilon_{1}}{N_{1}}\right),$$

where *N* is the maximal mRNA number, *A* is the steady state probability for the gene to be ON, ϵ is the gene switching speed (Equation (12)), and the subscript 1 indicates aimed post-treatment parameter values. A strategy to reduce $\sigma_{1}$ is to keep $N_{1}⪆1+\epsilon_{1}$ (or $k_{1}⪆f_{1}+h_{1}+\rho_{1}$ ), which ensures that $\sigma_{1}$ approaches the local maximum as $A_{1}\to 1$ . The reduction of $\sigma_{1}$ values depends on the mean number ${\langle n\rangle }_{1}$ desired, so that ${\langle n\rangle }_{1}\approx A_{1}\left(1+\epsilon_{1}\right)$ .

To enhance the previously ineffective treatments designs, we used the aforementioned optimization approach. The drug dose and interval of application are set to enable $\epsilon_{1}=91$ , which was found to be the value leading to the successful treatment design shown in D1 of Figure 2. Indeed, this resulted in the best response dynamics of the average number of RKIP mRNA’s as shown in I1 of Figure 3.

Figure 7A4 and B4 show the dynamics of response to enhanced treatment when pre-treatment RKIP gene transcription is governed by a slow switching promoter giving rise to bimodal probability distributions ( $\epsilon_{0}\leq 1$ ). The previously ineffective treatment design aimed at bimodal and table-shaped regimens are, respectively, shown by the green lines of A2 and B3 in Figure 2. The dynamics of response to the enhanced treatment designs are shown in Figure 7A2*,B3*. Both optimized responses resulted from similar changes in kinetic parameters that caused an increase in $A_{1}$ and a reduction in $N_{1}$ and have similar agendas (detailed in Appendix B).

Enhanced treatment design aiming for a pre-treatment fast switching gene shown in C4 and D4 of Figure 7. The initial probability distributions are table-shaped and quasi-Poissonian and have $\epsilon_{0}>1$ . The response dynamics to ineffective treatment designs are shown in C2 and D3 of Figure 2. The response to enhanced treatment with reduced heterogeneity and increased speed is shown in C2* and D3* of Figure 7. The enhanced treatment agendas were the same in both cases (see parameters and agendas in Appendix B). The value of ${\langle n\rangle }_{1}$ was reduced in comparison to the ineffective treatment design because of the decrease of $N_{1}$ not being compensated by the increase of $A_{1}$ .

The ineffective treatment design for an initially burst regimen of RKIP transcription shown in Figure 2E3 is enhanced. The resulting post-treatment distribution is a quasi-Poissonian. The dynamics of response to enhanced treatment design is shown, respectively, in E3* and E4 of Figure 7. This enhancement required the largest reductions in ${\langle n\rangle }_{1}$ in comparison to the ineffective design by increasing $A_{1}$ and decreasing $N_{1}$ . Note, however, the clear reduction in the standard deviations (heterogeneity) of the response. The parameters values and agenda applied are detailed in Appendix B.

## 4. Discussion

When the binary model for regulation of gene expression is considered, it is useful to characterize the pre-treatment gene expression regime in terms of $A_{0}$ , $\epsilon_{0}$ , and $N_{0}$ (see Equation (12)). Those rates will affect the pre-existing (and post-treatment) noise on the number of mRNAs, see Equations (13) and (14) (and Figure 3, Figure 4 and Figure 5 and Figure 7). Furthermore, the time of response to treatment also depends on $\epsilon_{0}$ , because the decaying to steady state regime of the average number of mRNAs and its variance have both ρ and ϵρ as their smallest rates (see Equation (15)). For ϵ>1 and <1 the time for the system to reach the steady state is, respectively, $\propto \rho^{-1}$ and $\propto {\left(\epsilon \;\rho \right)}^{-1}$ .

All trajectories of the average values shown in graphs of Figure 4 reach a maximal (Figure 4A2–E2) or cross the threshold (Figure 4F2–J2) after an approximately fixed interval. This is because the component $\propto e^{-\epsilon \rho t}$ of 〈n〉(t) is null as the treatment does not change the steady state probability for the gene to be ON (see Equation (A20)). Additionally, the noise on treatment response, measured in terms of the trajectories of 〈n〉(t)±σ(t) , is larger when we have the smaller values of the relative switching speed. Inspection of trajectories shown in A to D and F to I of Figure 3, Figure 4 and Figure 5 helps to identify those features.

The pre-treatment condition, which enables the fastest and least heterogeneous response takes place for higher values of the relative switching speed and for the probability for the gene to be ON being >0.1. D and I of Figure 3, Figure 4 and Figure 5 show that those are the conditions enabling the smallest differences between trajectories 〈n〉(t)±σ(t) . At this limit, the gene is behaving as a quasi-Poissonian source of transcripts [23]. As the value of the pre-treatment switching speed is reduced from toward two, we have an increase in the noise of the treatment response, though the speed of response does not increase significantly, as shown in C and H of Figure 3, Figure 4 and Figure 5.

The burst pre-treatment regime ( $\epsilon_{0}=10$ ) leads to the noisiest responses to treatment, although being among the fastest ones. The noisy response to treatment occurs partially because when we simulated treatment scenarios shown in E and J of Figure 3, Figure 4 and Figure 5, the value of the A(t) remained small. In that case, the dynamics would still remain in a transcriptional burst regime. The small increase in A(t) is because we assumed that the maximal tolerated dose of the drug responsible for increasing *f* would cause the probability of the ON state to be 10× larger, independent of its pre-treatment value. Such an assumption needs to be confirmed by experimental studies, and an alternative formulation might be proposed because of a lack of confirmation. B to E and G to J of Figure 3, Figure 4 and Figure 5 show that the time of response to treatment are all similar since the rates of decay to steady state are ≤ ρ for ϵ≤1 .

The slow relative switching speed pre-treatment condition (here, we set $\epsilon_{0}=0.1,\;1$ ) establishes a challenging regime to be approached. The response is slower and noisier if one takes the strategies considered here. A and F of Figure 3 and Figure 5 indicate that the increase in the average number of mRNA is the slowest when we have the minimal ϵ despite treatment increasing *f* will cause an increase of the relative switching speed.

In the high noise responses, the trajectories 〈n〉(t)−σ(t) do not cross the threshold. This indicates that, in a population of cells with the same regulatory conditions of expression of RKIP gene, the response to treatment is heterogeneous and may be insufficient. Therefore, the dosage and target need to be constructed to ensure a reduction in the response heterogeneity. This can be carried out by increasing the switching speed such that the gene expression will approach a quasi-Poissonian regime. Increasing the switching speed also has the benefit of reducing the time of the response to therapy.

The fractional effect of dose reduction of DETATANOate and 5-AzaC shown in Figure 6 indicates the difficulties of optimizing the agenda for pre-treatment regimes far from quasi-Poissonian. We highlight bimodal and burst regimes, where the responses need to have larger increases regarding the average mRNA levels. For that, one needs to target all effective kinetic rates of the stochastic binary model for gene expression. For the case of RKIP, one might also target Snail, BACH1, Sp1, CREB, and p300 repressors to change *h*, while a variety of miRNAs and their possible inactivators would modulate ρ [32].

The responses to enhanced treatments shown in Figure 7 (Figure 7A4–E4) indicate the potential of our approach to help on the design of low dose multi-drugs administered in pulses. The three qualitatively different pre-treatment regimens, namely slow bimodal, fast quasi-Poissonian, and burst, have distinguishable response curves. Those are resulting from the specificities of the enhanced treatment design aiming at keeping the average mRNA numbers and deviations (heterogeneity) above the threshold.

The synthesis of RKIP protein may have a non-linear dependence on the mRNA numbers because translation can be regulated by mechanisms, such as mRNA stability, translational control, and proteosomal degradation [78]. Hence, a theoretical approach considering translation might require the addition of two or more characteristic timescales depending on protein synthesis and degradation [90]. We emphasize that the amounts of RKIP mRNAs in our model are the net effect of the enhanced treatment, since its expression results from the interaction with a complex gene network. Our approach, however, provides the building blocks of gene networks [19] that can be used to understand the functioning of larger modules [14,15,16] and, hence, to further enhance treatment designs of cancer at metastatic stage.

5-AzaC and DETANONOate are non-specific drugs to increase RKIP, which may also interfere with additional biological processes taking place in tumoral or normal cells. The current study does not provide an insight on those problems and specific experimental designs are needed to approach these. Initially, one may consider simulations using pre-treatment conditions corresponding to the normal condition. In that case, it is fair to consider that the fraction of effect of reduced dose is smaller, if we assume that the drugs have a higher absorption in cancer cells. A more complex model for the dynamics of multiple genes is needed to investigate the overall effect of non-specific treatment designs. Alternatively, one might design treatments with higher specificity to further investigate the validity of our approach for simulating gene therapeutic designs.

Another possible application of our approach is on the investigation of therapeutic designs based on 5-AzaC and DETANONOate. For example, 5-AzaC promotion of DNA demethylation could be used to regulate the expression of genes involved in reverting adult stem cell ageing [91], while its inhibitory properties of DNA methyltransferase has the potential for regenerating mature mammalian inner ear hair cells [92]. NO donors as DETANONOate are involved in many cell processes, such as vasodilation, neurotransmission, macrophage-mediated immunity, and anti-inflammatory responses [93]. One application of our approach is to help in elucidating the mechanisms of those therapeutic designs.

To further improve our approach, one needs to perform a data-based validation of our model. One possible experiment would involve the decrease of RKIP levels using a siRNA approach to simulate different levels of activation of the RKIP gene. This would be a more specific and precise way to interfere in the RKIP pathway compared with using 5-AzaC. We could make different levels of RKIP inhibition by siRNA and see if the modeling can predict MEK and ERK phosphorylation levels, the expression of MAPK-induced genes, and the consequence of these activations in cell migration, proliferation, and invasion. The siRNA approach is not able to check the OFF state of the RKIP gene; however, it is possible to use a CRISPR/CAS9 system to eliminate the RKIP gene, and then have the OFF state information. Inference methods would be employed on the estimation of parameter values and RKIP mRNA numbers.

Those experiments would help to understand how environmental information is processed by cells by investigating how regulation of expression of RKIP gene affects its relative downstream kinases. In recent work, we demonstrated that the slow switching speed regime maximizes the mutual information between the number of transcripts and promoter activity [34]. This regime also coincides with conditions of highly heterogeneous and slow response to treatment. Hence, we may ask whether it is relevant for a master regulatory gene to transmit information about its own promoter state and in which cellular context, namely, in a cancerous or healthy one. The answer to this question is important because it helps us to understand the role of noise and the conditions under which its reduction is desirable [33] as it happens when we have a negatively self-regulating gene.

## 5. Conclusions

In this manuscript, we present a stochastic binary model for transcription of the RKIP gene with treatment-induced time-dependent kinetic rates. The exact solutions of this model are approximated using the exact solutions of the equivalent model with stationary kinetic rates. This is the simplest exactly solvable model to describe the regulated transcription of the RKIP gene. This enables us to simulate the effects of the application of a drug that changes one (or multiple) kinetic parameters participating in the regulation of RKIP gene.

To demonstrate the usefulness of our approach, we simulated three scenarios in which we aimed to increase the number of RKIP mRNAs by increasing the: i. OFF to ON switching rate using DETANONOate; ii. synthesis rate using 5-AzaC; and iii. both rates of a gene using both drugs together. We showed that treatment response speed and heterogeneity depended on the pre-treatment state of the gene. Then, we presented an enhanced treatment design that ensured reduced heterogeneity and time of response. In addition to being useful to inspect treatment designs, the response to treatment may be used for inference of the kinetic constants of a given gene in a synthetic system.

## Figures and Tables

**Table 1:** The fractions $\xi_{a}$ and $\xi_{b}$ of the maximal tolerated dose for treatment agendas of the pre-treatment conditions in Figure 5.

Graph	Sequence of Number of Doses × Fraction for		Cumulative Reduction in	
	$\xi_{a}$	$\xi_{b}$	$\xi_{a}$	$\xi_{b}$
F3	3×0.9 ; 4×0.8 ; 1×0.5	5×0.9 ; 10×0.8 ; 5×0.7	20%	20%
G3	3×0.8 ; 1×0.7 ; 2×0.5	5×0.8 ; 10×0.75	32%	23%
H3	3×0.8 ; 1×0.7 ; 2×0.5	5×0.8 ; 10×0.75	32%	23%
I3	4×0.7 ; 1×0.6 ; 1×0.5	15×0.7	35%	30%
J3	3×0.7 ; 1×0.65 ; 2×0.6	5×0.7 ; 5×0.65 ; 5×0.6	29%	35%

## Data Availability

The codes can be provided under request to the corresponding author.

## Appendix A. Formulae

### Appendix A.1. Piecewise Approximation for Time-Dependent Kinetic Parameters

We denote by $E_{i}$ the error of the piece-wise approximation for the decay of the drug effect on a given rate within an interval $\left[t_{i},\;t_{i}+\delta_{i}\right)$ . We compute the error as the absolute value of the difference between area of the rectangle with base $\delta_{i}>0$ and height $e^{-\lambda t_{i}}$ , and the area under the exponential function $e^{-\lambda t}$ within the interval $\left[t_{i},\;t_{i}+\delta_{i}\right]$ , namely:

(A1)
$$E_{i}=\delta_{i}e^{-\lambda t_{i}}-\int_{t_{i}}^{t_{i}+\delta_{i}}e^{-\lambda t^{\prime }}dt^{\prime }=\frac{e^{-\lambda t_{i}}}{\lambda }\left(\lambda \delta_{i}-1+e^{-\lambda \delta_{i}}\right).$$

Note that $E_{i}$ is positive definite.

Once the error $E_{i}$ on an interval is set, we may solve a transcendental equation $-\lambda \delta_{i}+1+\lambda e^{\lambda t_{i}}E_{i}=e^{-\lambda \delta_{i}}$ for $\delta_{i}$ using some numerical procedure. In this manuscript, we set $E_{i}=10^{-4}$ for all subintervals along $\left[0,t_{\max}\right]$ .

### Appendix A.2. System of Coupled ODEs Governing the Moments of the Distribution

Here, we describe the process for obtaining the time-dependent solutions for 〈n〉(t) and $\sigma^{2}\left(t\right)$ given at Equations (15) and (16). This is carried out considering the kinetic rates to be constant in the master Equations (10) and (11), which become:

(A2)
$$\begin{array}{ccc} \frac{d\alpha_{n}}{dt} & = & k\left(\alpha_{n-1}-\alpha_{n}\right)+\rho \left[\left(n+1\right)\alpha_{n+1}-n\alpha_{n}\right]-h\alpha_{n}+f\beta_{n}, \end{array}$$

(A3)
$$\begin{array}{ccc} \frac{d\beta_{n}}{dt} & = & \rho \left[\left(n+1\right)\beta_{n+1}-n\beta_{n}\right]+h\alpha_{n}-f\beta_{n}. \end{array}$$

The master equations above govern the dynamics of the joint probability distribution $\Pi (\alpha_{n},\beta_{n})$ . This distribution has two marginal probability distributions: *i.* the probability of finding *n* mRNAs, independently of the promoter state, is denoted by $\phi_{n}$ and given by:

(A4)
$$\phi_{n}\left(t\right)=\alpha_{n}\left(t\right)+\beta_{n}\left(t\right);$$

*ii.* The probability of finding the promoter state as ON or OFF is, respectively, denoted by A(t) and B(t) and given by:

(A5)
$$A\left(t\right)=\sum_{n=0}^{\infty }\alpha_{n},\;\;\mathrm{and}\;\;B\left(t\right)=\sum_{n=0}^{\infty }\beta_{n}.$$

In what follows, we describe how the generating functions technique can be used to obtain explicit expressions for these probabilities and associated moments.

The master Equations (A2) and (A3) can be viewed as an infinite system of coupled ordinary differential equations whose exact solution is usually difficult to obtain. In solving it, we may employ the generating functions technique, which considers the probabilities $\alpha_{n}\left(t\right)$ and $\beta_{n}\left(t\right)$ as coefficients of a Taylor expansion of an analytic function, namely:

(A6)
$$\alpha \left(z,t\right)=\sum_{n=0}^{\infty }\alpha_{n}\left(t\right)z^{n}\;\;\;\beta \left(z,t\right)=\sum_{n=0}^{\infty }\beta_{n}\left(t\right)z^{n}.$$

The application of the definitions above enables one to transform Equations (A2) and (A3) into a pair of partial differential equations (PDEs) on variables (z,t) , namely:

(A7)
$$\begin{array}{ccc} \frac{\partial \alpha }{\partial t} & = & \left(z-1\right)\left(k\alpha -\rho \frac{\partial \alpha }{\partial z}\right)-h\alpha +f\beta , \end{array}$$

(A8)
$$\begin{array}{ccc} \frac{\partial \beta }{\partial t} & = & -\rho \left(z-1\right)\frac{\partial \beta }{\partial z}+h\alpha -f\beta . \end{array}$$

This pair of coupled PDEs is obtained by first multiplying both sides of Equations (A2) and (A3) by $z^{n}$ , and then, by application of the operator $\sum_{n=0}^{\infty }$ .

Here, we are interested in understanding the dynamics of both 〈n〉(t) and $\sigma^{2}\left(t\right)$ . For that, we need to recover the system of ODEs governing the *p*-th order moments of the distribution $\Pi (\alpha_{n},\beta_{n})$ , where p=1,2,… . This is done by applying the ${\left(z\frac{\partial }{\partial z}\right)}^{p}$ on Equations (A7) and (A8) and, on the resulting expression, setting z=1 . This procedure is proposed because the *p*-th order moment may be recovered from the generating functions as follows:

(A9)
$$\begin{array}{c} \langle n_{\alpha }^{p}\rangle =\sum_{n=0}^{\infty }\alpha_{n}n^{p}={\left.\left[{\left(z\frac{\partial }{\partial z}\right)}^{p}\alpha \left(z,t\right)\right]\right|}_{z=1}, \end{array}$$

(A10)
$$\begin{array}{c} \langle n_{\beta }^{p}\rangle =\sum_{n=0}^{\infty }\beta_{n}n^{p}={\left.\left[{\left(z\frac{\partial }{\partial z}\right)}^{p}\beta \left(z,t\right)\right]\right|}_{z=1}. \end{array}$$

(A11)
$$\begin{array}{c} \langle n^{p}\rangle =\sum_{n=0}^{\infty }\phi_{n}n^{p}={\left.\left[{\left(z\frac{\partial }{\partial z}\right)}^{p}\phi \left(z,t\right)\right]\right|}_{z=1}. \end{array}$$

Here, ϕ(z,t)=α(z,t)+β(z,t) is the generating function of the marginal probability of finding *n* mRNAs, $\phi_{n}$ . Hence, $\langle n^{p}\rangle$ is the *p*-th order moment on the number of mRNAs.

The expressions of 〈n〉(t) and $\sigma^{2}\left(t\right)$ are obtained after solving the following system composed of linear non-homogeneous coupled ODEs:

(A12)
$$\begin{array}{ccc} & & \frac{dA}{dt}+\left(f+h\right)A=f, \end{array}$$

(A13)
$$\begin{array}{ccc} & & \frac{d\langle n\rangle }{dt}+\rho \langle n\rangle =kA, \end{array}$$

(A14)
$$\begin{array}{ccc} & & \frac{d\langle n_{\alpha }\rangle }{dt}+\left(f+h+\rho \right)\langle n_{\alpha }\rangle =kA+f\langle n\rangle , \end{array}$$

(A15)
$$\begin{array}{ccc} & & \frac{d\langle n^{2}\rangle }{dt}+2\rho \langle n^{2}\rangle =kA+\rho \langle n\rangle +2k\langle n_{\alpha }\rangle , \end{array}$$

where we omitted the time-dependence of *A*, 〈n〉 , $\langle n_{\alpha }\rangle$ , and $\langle n^{2}\rangle$ .

The above system of ODEs was obtained as follows. 1. Equation (A12): at Equation (A7) we set, in the following order, β=ϕ−α , z=1 , $\alpha \left(1,t\right)=A\left(t\right)$ , and $\phi \left(1,t\right)=1$ . 2. Equation (A13): in following order, we sum Equations (A7) and (A8), set β=ϕ−α , apply the operator $\frac{\partial }{\partial z}$ , evaluate the resulting equation at z=1 , and use the identities $\alpha \left(1,t\right)=A\left(t\right)$ , and ${\left.\frac{\partial \phi }{\partial z}\right|}_{z=1}=\langle n\rangle$ . 3. Equation (A14): at Equation (A7) we set, in the following order, β=ϕ−α , apply the operator $\frac{\partial }{\partial z}$ , evaluate the resulting equation at z=1 , and use the identities $\alpha \left(1,t\right)=A\left(t\right)$ , ${\left.\frac{\partial \phi }{\partial z}\right|}_{z=1}=\langle n\rangle$ , and ${\left.\frac{\partial \alpha }{\partial z}\right|}_{z=1}=\langle n_{\alpha }\rangle$ . 4. Equation (A15): in following order, we sum Equations (A7) and (A8), set β=ϕ−α , apply the operator $\frac{\partial^{2}}{\partial z^{2}}$ , evaluate the resulting equation at z=1 , and use the identities $\alpha \left(1,t\right)=A\left(t\right)$ , ${\left.\frac{\partial \phi }{\partial z}\right|}_{z=1}=\langle n\rangle ,{\left.\frac{\partial \alpha }{\partial z}\right|}_{z=1}=\langle n_{\alpha }\rangle$ , and ${\left.\frac{\partial^{2}\phi }{\partial z^{2}}\right|}_{z=1}=\langle n^{2}\rangle -\langle n\rangle$ .

### Appendix A.3. Steady State Values of the Moments of the Distribution

To obtain the steady state solutions of the system of EDOs of Equations (A12)–(A15) one may set $A\to A_{s}$ , $\langle n\rangle \to {\langle n\rangle }_{s}$ , $\langle n_{\alpha }\rangle \to {\langle n_{\alpha }\rangle }_{s}$ , and $\langle n^{2}\rangle \to {\langle n^{2}\rangle }_{s}$ , where the index *s* indicates these quantities at the steady state limit, when they become constant. Hence, the derivatives are null and one may solve the resulting system of equations. Equation (A12) is solved for $A_{s}$ , which is replacedinthe next, and so on. This procedure will result in

(A16)
$$\begin{array}{ccc} A_{s} & = & \frac{f}{f+h}, \end{array}$$

(A17)
$$\begin{array}{ccc} {\langle n\rangle }_{s} & = & A_{s}N \end{array}$$

(A18)
$$\begin{array}{ccc} {\langle n_{\alpha }\rangle }_{s} & = & A_{s}N\frac{1+A_{s}\epsilon }{1+\epsilon }, \end{array}$$

(A19)
$$\begin{array}{ccc} {\langle n^{2}\rangle }_{s} & = & A_{s}N\left(1+N\frac{1+A_{s}\epsilon }{1+\epsilon }\right). \end{array}$$

### Appendix A.4. Coefficients of Equations (15) and (16)

(A20)
$$\begin{array}{ccc} Y & = & N\frac{\left(A_{0}-A_{s}\right)}{1-\epsilon }, \end{array}$$

(A21)
$$\begin{array}{ccc} V & = & {\langle n\rangle }_{0}-Y-{\langle n\rangle }_{s}\;, \end{array}$$

(A22)
$$U_{1}=U-2Y{\langle n\rangle }_{s},\;\;\;W_{1}=W-2YV,\;\;\;X_{1}=X-V^{2},$$

(A23)
$$\begin{array}{ccc} U & = & Y\left[1+2N\frac{1-\epsilon \left(1-A_{s}\right)}{2-\epsilon }\right], \end{array}$$

(A24)
$$\begin{array}{ccc} W & = & \frac{2N}{1-\epsilon }\left[N\left(1-A_{s}\right)\frac{\epsilon \;\left(A_{s}-A_{0}\right)-A_{0}}{\epsilon +1}-A_{s}{\langle n\rangle }_{0}+{\langle n_{\alpha }\rangle }_{0}\right], \end{array}$$

(A25)
$$\begin{array}{ccc} X & = & {\langle n^{2}\rangle }_{0}-{\langle n^{2}\rangle }_{s}-U-\left(1+2{\langle n\rangle }_{s}\right)V-W\;. \end{array}$$

### Appendix A.5. Exact Formulas for A(t), 〈n_α_〉(t), and 〈n^2^〉(t)

The exact time-dependent solutions of the system of EDOs of Equations (A12)–(A15) can be obtained using the method of variation of the constant (see Section 9.2 of [94]). For that, one may solve Equation (A12) for A(t) , insert it in the next equation, and so on. The resulting solutions are:

(A26)
$$A\left(t\right)=A_{s}+\left(A_{0}-A_{s}\right)e^{-\left(h+f\right)t}\;,$$

(A27)
$$\langle n\rangle \left(t\right)={\langle n\rangle }_{s}+Y\;e^{-\epsilon \rho t}+V\;e^{-\rho t},$$

(A28)
$$\langle n_{\alpha }\rangle \left(t\right)={\langle n_{\alpha }\rangle }_{s}+Re^{-\left(h+f\right)t}+Se^{-\rho \;t}+Te^{-\left(f+h+\rho \right)t}\;,$$

(A29)
$$\langle n^{2}\rangle \left(t\right)={\langle n^{2}\rangle }_{s}+Ue^{-\left(h+f\right)t}+\left(1+2{\langle n\rangle }_{s}\right)Ve^{-\rho \;t}+We^{-\left(f+h+\rho \right)t}+Xe^{-2\;\rho \;t}\;,$$

where

(A30)
$$\begin{array}{ccc} R & = & Y\left[1-\epsilon \left(1-A_{s}\right)\right]\;, \end{array}$$

(A31)
$$\begin{array}{ccc} S & = & A_{s}{\langle n\rangle }_{0}+{\langle n\rangle }_{s}\frac{A_{s}\epsilon -A_{0}}{1-\epsilon }\;, \end{array}$$

(A32)
$$\begin{array}{ccc} T & = & {\langle n_{\alpha }\rangle }_{0}-{\langle n_{\alpha }\rangle }_{s}-R-S\;. \end{array}$$

### Appendix A.6. Steady State Probabilities of Finding n Gene Products

The steady state probability distributions presented in this manuscript were obtained from the exact solutions of the stochastic binary model for regulation of gene expression presented in [19,95]. Here, we denote the steady state marginal probabilities of finding *n* gene products by ${\tilde{\phi }}_{n}$ . It is defined in terms of the probabilities of finding the gene ON or OFF when *n* gene products are found inside the cell, which are denoted by ${\tilde{\alpha }}_{n}$ or ${\tilde{\beta }}_{n}$ . Hence we write ${\tilde{\phi }}_{n}={\tilde{\alpha }}_{n}+{\tilde{\beta }}_{n}$ , which is given by:

(A33)
$${\tilde{\phi }}_{n}\left(A_{s},\epsilon ,N\right)=\frac{N^{n}}{n!}\frac{{\left(A_{s}\epsilon \right)}_{n}}{{\left(\epsilon \right)}_{n}}M\left(A_{s}\epsilon +n,\epsilon +n,-N\right),$$

where ${\left(a\right)}_{n}\equiv a\left(a+1\right)\cdots \left(a+n-1\right)$ is the Pochhammer symbol and $M\left(a,b,z\right)=\sum_{m=0}^{\infty }\frac{{\left(a\right)}_{m}}{{\left(b\right)}_{m}}\frac{z^{m}}{m!}$ is the KummerM function ([96], p. 503).

## Appendix B. Treatment Parameters and the Agenda of Doses for Enhanced Treatment Designs

The following table shows the treatment parameters used to obtain the graphs of Figure 7. The post-treatment kinetic rates, $\left(k_{1},\;\rho_{1},\;f_{1},\;h_{1}\right)$ , and distributions parameters, $\left(N_{1},\;A_{1},\;{\langle n\rangle }_{1},\;\sigma_{1}\right)$ , where set to enable the enhanced treatment designs. The number of doses is represented by $n_{i}$ and the time interval between them by $\tau_{i}$ , where *i* indicates the aimed kinetic rate. For all treatments, $\epsilon_{1}=91$ , $n_{f}=3$ and $\tau_{f}=10$ .

**Graph**	$\left(k_{1},\;\rho_{1},\;f_{1},\;h_{1}\right)$	$\left(N_{1},\;A_{1},\;{\langle n\rangle }_{1},\;\sigma_{1}\right)$	$\left(n_{k},\;n_{\rho },\;n_{h}\right)$	$\left(\tau_{k},\;\tau_{\rho },\;\tau_{h}\right)$
A4	(112,0.83,58,18)	(135,0.76,103,11.8)	(8,30,30)	(4,1,1)
B4	(91,0.67,40,21)	(136,0.65,88,11.6)	(8,30,15)	(4,1,2)
C4/D4	(44,0.42,23,15)	(106,0.6,63.6,9.6)	(30,30,30)	(1,1,1)
E4	(235,1.17,23,83)	(201,0.22,44,10.9)	(8,10,10)	(4,3,3)
